# The qualitative study of intentional self-harm in Thailand: Focusing on predisposing child-rearing environments and self-harm cessation

**DOI:** 10.3389/fpsyg.2023.957477

**Published:** 2023-03-28

**Authors:** Nida Limsuwan, Anyamanee Lantomrattana, Thanavadee Prachason, Thanita Tantrarungroj, Passaporn Lorterapong, Masatha Thongpan, Punjaporn Waleeprakhon

**Affiliations:** Department of Psychiatry, Faculty of Medicine Ramathibodi Hospital, Mahidol University, Ratchathewi, Bangkok, Thailand

**Keywords:** self-harm, suicide, suicidal behaviors, NSSI, qualitative study

## Abstract

**Introduction:**

Intentional self-harm in adolescents and young people, including both suicidal behaviors and non-suicidal self-injury (NSSI), is a serious issue in mental health systems. However, the majority of studies on self-harm in adolescents and young people focused on a quantitative methodology which might have limitations in explaining this complex phenomenon of intentional self-harm. Therefore, this study aimed to describe the subjective experiences of adolescents and young people who presented with intentional self-harm in order to provide a better understanding of this behavioral phenomenon.

**Methods:**

This is an exploratory qualitative study that uses phenomenological processes and thematic analysis. Twenty subjects aged 13–29 years were included in this study.

**Results:**

The results revealed six themes regarding predisposing child-rearing environments and nine themes regarding factors related to the cessation of intentional self-harm. Moreover, it demonstrated the important functions of self-harm as an intrapersonal strategy for emotional regulation.

**Discussion:**

In conclusion, this study underscored the importance of understanding the developmental and cessation pathways of these complex behaviors.

## Introduction

Intentional self-harm in adolescents and young people, including both suicidal behaviors and nonsuicidal self-injury (NSSI), is a serious issue in mental health systems. It has profound impacts on individuals, families, communities, and societies. In 2019, the World Health Organization (WHO) estimated that 703,000 people died by suicide worldwide and reported that self-harm was the fourth leading cause of death among adolescents and young adults aged 15–29 years (World Health Organization, 2019). Recently, the meta-analysis showed that the global lifetime and 12-month prevalence of deliberate self-harm in children and adolescents was 13.7 and 14.2%, respectively (Lim et al., 2019). In general, girls were more likely to self-harm than boys (risk ratio 1.7) (Gillies et al., 2018). In addition, the mean age of starting self-harm was 13 years, and cutting was the most common method (45%) (Gillies et al., 2018).

Despite intentions, suicidal attempts and NSSI share some clinical presentations, such as depressive symptoms, hopelessness, and low self-esteem (Hamza et al., 2012; Hawton et al., 2012). In addition, some studies showed a complex relationship between these two conditions; for example, NSSI can predict subsequent suicidal behaviors (Castellvi et al., 2017; Knorr et al., 2019; Mars et al., 2019). Some clinicians viewed these conditions as two different manifestations of the same behavioral spectrum (Hamza et al., 2012). Unsurprisingly, some people have dynamically presented with these two different conditions of intentional self-harm over time.

Self-harm is a complex interplay between biological, psychological, psychiatric, social, and cultural factors. Some experts explained this behavioral phenomenon based on the diathesis–stress model; for instance, predisposing personality traits such as impulsivity combine with negative life events, including child maltreatment and maladaptive parenting, to develop self-destructive behaviors across the lifespan (Hawton et al., 2012). Moreover, several studies emphasized on social transmission and the contagious effect of self-harm, especially in specific populations such as adolescents (Cheng et al., 2014). Furthermore, the Internet and social media play a bigger part in providing dramatic narratives and details of methods used in self-harm than traditional media that might encourage these behaviors (Patchin and Hinduja, 2017; Biernesser et al., 2020; Wang et al., 2020).

However, the majority of studies on self-harm in adolescents and young people focused on a quantitative methodology. It was conducted in Western countries, which might have limitations in explaining this complex phenomenon of intentional self-harm. Cultural differences and different social contexts potentially affect emotional and behavioral expression. In addition, attitudes, beliefs, and perspectives regarding intentional self-harm might be different between Western and Eastern cultures. As a result, this present study aimed to describe the subjective experiences of adolescents and young people who presented with intentional self-harm, including both suicidal behaviors and NSSI in Thailand, in order to provide a better understanding of this behavioral phenomenon, especially predisposing child-rearing environments and how people stop these behaviors.

## Materials and methods

This was an exploratory qualitative study that used phenomenological processes and thematic analysis. Qualitative methodology was used to identify and describe participants' perceptions and experiences regarding intentional self-harm. Twenty in-depth interviews were conducted with adolescents and young people who presented with intentional self-harm.

### Sampling

Purposeful sampling, which was widely used in qualitative research, was performed in this study. The adolescents and young people who presented with intentional self-harm were recruited from the psychiatric outpatient department at the Ramathibodi Hospital, Mahidol University, Bangkok, Thailand. The inclusion criteria were as follows: (1) age 13–29 years, (2) history of intentional self-harm, (3) ability to communicate and express their experience and perception in Thai, and (4) currently mild psychiatric symptoms. The subjects who had moderate to severe psychiatric symptoms such as psychotic symptoms, acute manic symptoms, and actively suicidal ideation were excluded.

### Data collection

All psychiatrists in the department were informed regarding the research protocol, and they made suggestions and introduced their own clients who probably matched the study protocol to the research team. Afterward, the research team contacted the participants, performed the primary assessments, and informed consent was obtained from both adolescents and their parents in case of participants under the age of 18.

Subsequently, the research team members arranged face-to-face semi-structured interviews for each participant. The interviewers were experienced psychiatrists, including three general psychiatrists for adult participants and four child psychiatrists for participants aged 12–18 years. Each interviewing session performed by two interviewers included the primary interviewers, who mainly asked semi-structured interview questions (Table 1), and the secondary interviewers, who mainly observed non-verbal expressions, recorded and added on more questions if they were curious to find out something during the interviews. In general, the interview duration ranged from 60 to 90 min. They were audio-recorded and transcribed verbatim. All the interviews were conducted between May 2020 and March 2021. Moreover, the researcher meetings were arranged every 10 sessions of the interview to indicate whether the data was saturated.

**Table 1:** Focus areas of semi-structured interviewing.

**Focus areas**	**Samples of questions**
**Self-harm behaviors**	• Can you tell me about methods you used for self-harm? • When did you start to (method) yourself? • Would you please tell me about your motivations for self-harm? • If you have ever stopped self-harm, how did that happen?
**Antecedents**	• Before you did self-harm, did you feel stressed about anything? • Before you did self-harm, did you have any academic pressure? • Have you ever learned how to do self-harm from social media or other media?
**Consequences**	• After you did the self-harm, how did you feel? • After you did the self-harm, would you please tell me about the reactions from your parents and friends? • Did you get any unwanted consequences after your self-harm?

### Ethical standards

This present study procedure was approved by the Ethics Committee of Ramathibodi Hospital, Mahidol University. All participants and their parents (in the case of participants under the age of 18) gave written informed consent for the research in accordance with the Declaration of Helsinki. In addition, all interview records and documents have been anonymized, and the datasets were non-personally identifiable.

### Analysis of results

The transcribed interviews were analyzed by using a thematic analysis. The analytic team included two child psychiatrists (AL, NL) and a general psychiatrist with research methodology skills (TP). First, initial coding was independently generated by the researchers who performed each interview session. Second, the data were read several times by the analytic team, who closely examined the original data in order to become familiar with the data. Third, the analytic team examined the initial coding coupled with the original data to identify themes and perform the axial coding by grouping and organizing the data into meaningful categories. Occasionally, the analytic team made the interpretation and conceptualization of the initial coding and the data in order to define the relevant themes. Finally, the labeling of themes was reviewed and discussed among all the researchers to reach an agreement. Investigator triangulation was used to provide multiple observations, perspectives, and conclusions among all researchers.

## Results

Twenty subjects, 16 women and 4 men, were included in this study. The age of participants ranged from 13 to 29 years, and the age at the onset of self-harm ranged from 4 to 21 years. Regarding types of intentional self-harm, more than half of the participants (*n* = 12, 60%) reported both NSSI and suicidal behaviors; however, the rest (*n* = 8, 40%) conducted only NSSI. Therefore, there was no suicide-only subgroup in this present study. The most common method used for suicidal acts was drug overdose (*n* = 7, 35%), and for NSSI, it was cutting (*n* = 19, 95%). Most of the participants (*n* = 16, 80%) stopped their self-harm behaviors for at least 1 month; on the other hand, four participants (20%) reported ongoing self-harm. The mean duration of self-harm cessation was 5.3 months (min = 1 month, max = 12 months). In addition, the most common psychiatric diagnosis among all participants was major depressive disorder (*n* = 11, 55%). The basic characteristics of participants are summarized in Table 2. In addition, we found that large numbers of the participants experienced psychosocial adversity, as summarized in Table 3.

**Table 2:** Basic characteristics.

**Order**	**Age at interview (Year)**	**Sex**	**Age at onset of self-harm (Year)**	**Duration of cessation**	**Psychiatric diagnoses**	**Types of intentional self-harm**	**Methods**
1	28	Female	10	Ongoing	MDD, Anxiety disorder	NSSI + SA	Cutting, pinching, toxic ingestion, strangulation, jumping off building, car collision
2	23	Female	21	7 mo.	MDD	NSSI	Cutting, pinching, hitting herself
3	13	Female	12	1 mo.	Adjustment disorder	NSSI	Drug overdose
4	14	Female	13	Ongoing	ADHD, History of MDD	NSSI	Cutting, pinching, hitting object
5	14	Female	13	1 yr.	Bipolar I disorder	NSSI	Cutting, pinching, hitting object
6	16	Female	15	6 mo.	MDD	NSSI	Cutting
7	20	Female	15	1 yr.	MDD, ADHD	NSSI	Cutting, hitting herself, strangulation, drug overdose, car collision
8	17	Male	16	1 mo.	Dysthymia, ADHD	NSSI	Cutting, hitting object, scratching
9	18	Female	4	1 yr.	Dysthymia	NSSI + SA	Cutting, pinching, scratching, rubbing skin against rough surface, hanging, jumping off building
10	18	Female	8	Ongoing	MDD, PTSD	NSSI + SA	Cutting, hair pulling, toxic ingestion, hanging
11	15	Female	5	1 mo.	MDD	NSSI + SA	Breath holding, cutting, pinching, drug overdose
12	18	Female	16	1 yr.	MDD, Social anxiety	NSSI	Cutting, pinching
13	18	Female	14	5 mo.	Bipolar disorder	NSSI + SA	Cutting, pinching, hitting object, jumping off building
14	18	Female	15	1 mo.	Dysthymia	NSSI + SA	Cutting, hitting object, drug overdose
15	20	Male	19	1 mo.	MDD	NSSI + SA	Cutting, drug overdose
16	18	Female	16	3 mo.	MDD	NSSI + SA	Cutting, drug overdose
17	29	Male	11	3 mo.	MDD	NSSI + SA	Cutting, hitting himself, intentionally let others hit him, strangulation, toxic ingestion, jumping off building
18	14	Female	12	1 mo.	Bipolar II disorder	NSSI + SA	Cutting, drug overdose
19	14	Female	13	Ongoing	Dysthymia, PTSD	NSSI + SA	Cutting, hitting object, hanging, toxic ingestion, jumping off building
20	19	Male	12	6 mo.	MDD, ADHD	NSSI + SA	Cutting, hitting object, interfering wound healing processes, strangulation

ADHD, attention deficit hyperactivity disorder; MDD, major depressive disorder; PTSD, post-traumatic stress disorder; NSSI, non-suicidal self-injury; SA, suicidal act.

**Table 3:** History of psychosocial adversity and vulnerability.

**History of psychosocial adversity and vulnerability**	**Frequency (n)**	**Percentage (%)**
Bully victims	13	65
Multiple placements	6	30
Residences	3	15
Schools	3	15
Divorced families	6	30
Loss and grief (death of parents/attachment figures)	6	30
Maltreatment	9^*^	45
Sexual abuse	4	20
Emotional or verbal abuse	4	20
Physical abuse	6	30
Neglect	5	25

* 6 participants experienced more than 1 types of maltreatment.

Regarding the functions of self-harming behaviors, we divided them into two main groups: intrapersonal functions and interpersonal functions. Focusing on intrapersonal functions, all participants (*n* = 20, 100%) reported that they used self-harm as a strategy for emotional regulation, including a way to escape unwanted mental states (*n* = 20, 100%) and a way to induce wanted mental states (*n* = 12, 60%). Moreover, some participants (*n* = 5, 25%) described self-harm as a way to self-punishment. Interestingly, the minority of participants reported other intrapersonal functions, including a substitution of suicide or a way to relate to death (*n* = 3, 15%), response to urges to self-harm (*n* = 1, 5%), and coping with unsafe feeling (*n* = 1, 5%). Focusing on interpersonal functions, four participants (*n* = 4, 20%) reported self-harm as a way to communicate with others, and six participants (*n* = 6, 30%) described self-harm as a way to influence/manipulate others, including calling for attention. In addition, most participants (*n* = 17, 85%) mentioned multiple functions of self-harm, not only one function. However, intrapersonal functions were obviously demonstrated as dominant functions in this study population (Table 4).

**Table 4:** Functions of intentional self-harm.

**Functions**	**Frequency (n)**	**Percentage (%)**
• **Intrapersonal**		
Emotional regulation	20	100
- A way to escape unwanted mental states	20	100
- A way to induce wanted mental states	12	60
Self-punishment	5	25
Substitution of suicide or a way to relate to death	3	15
Response to urges to self-harm	1	5
Coping with unsafe feeling	1	5
• **Interpersonal**		
A way to influence/manipulate others	6	30
A way to communicate to others	4	20

In addition, we reported six themes regarding predisposing child-rearing environments and nine themes regarding factors related to the cessation of intentional self-harm, which were divided into two groups: internal and external factors.

### Predisposing child-rearing environments

#### Lack of emotional responsiveness/emotional neglect (n = 13)

Lack of emotional responsiveness or emotional neglect can happen in many situations. For instance, parents have not provided enough quality time for their children because they are busy with earning a living or their own emotional problems. Several participants described a silent atmosphere in their families and distance in terms of emotional involvement and communication. In addition, some participants reported that their parents/caregivers were naturally quiet or had poor communication skills.

“I feel that our family has problems when we don't talk to each other at all.” (Case 4)

“Dad covers my expenses, but never supports me emotionally. He never has time.” (Case 9)

“There were times when my aunt (who had taken care of me) was mad, and we didn't talk for almost a year. I have no idea whom she was angry at, but she talked to no one.” (Case 12)

Moreover, one participant mentioned he was preoccupied with academic and sports training activities arranged by his parents; as a result, he did not have time to connect emotionally with his parents.

“I was preoccupied with learning. After school, I always attended extra-tutorial sessions, then I had to practice swimming until very late at night. I have rarely seen my parents.” (Case 17)

Consequently, the emotional distance between parents/caregivers and children was founded in several cases. Some participants described that they had to stand by themselves in daily life activities, or even through difficult times since they were young, without emotional support from caregivers. Lack of warmth in the family atmosphere and loneliness were mentioned.

“Home is not home. Home is a place … not nice to live. We separately live.” (Case 1)

“I always live by myself.” (Case 8)

“I feel that … he is just dad. I don't feel close to dad.” (Case 13)

#### Negativity, criticism, and harsh punishment (*n* = 12)

Several participants reported negativity and criticism in their interaction with adults, which led to emotional pain and a negative sense of self. Blaming, looking down on, and insulting were described by several participants. Moreover, harsh punishment was described in several contexts, including parenting, sports training, and academic activities. Some participants described that they did not understand why the adults severely reacted with violence to their minor mistakes. In addition, some participants reported that they were punished because they could not perform as well as the adults wanted them to achieve.

“He always says things that make me feel bad. It isn't forcing, but it makes me feel like I have never done anything right. It seems like I'm not good enough. Whatever I do will never be good enough.” (Case 7)

“I just forgot my shoes, and then dad pulled my hair and hit me at the back.” (Case 10)

“He has repeated and repeated … the way he talks will make me hurt, but he chose to talk this way.” (Case 19)

#### High academic expectations (n = 9)

Some participants reported high academic expectations originating from their families and their stress on academic performance, including getting good grades, getting into top-ranking schools/universities, and working in respected/prestigious professions such as medicine and engineering. Although these expectations and attitudes are generally initiated by parents or adults in families, some participants described that these expectations were subsequently internalized and became their own standards and expectations.

“It was really stressful about the schoolwork because mom pushed me too hard. Mom was like ‘Hey, you studied this already! You're supposed to get it right.”' (Case 4)

“I had to get all A's. Can't get anywhere lower than 18 out of 20.” (Case 7)

#### Comparison with siblings (n = 5)

Five participants mentioned about their sibling rivalry and the patterns of communication in which their parents/caregivers often compared them with their siblings. These comparisons became a source of pressure to be similar, different, or better than their siblings, especially in terms of achievement or success.

“I want to be great like my sister. I'm a loser.” (Case 10)

“Since I was young, he would expect me to do better than my sister. It was such a pressure.” (Case 20)

#### Superficial responsiveness (n = 4)

Some parents/caregivers generally responded to their children; however, their responses were superficial in terms of affective involvement. In addition, these responses lacked content that provided a sense of caring and genuine understanding of the children's situations and difficulties; as a result, the lack of depth in communication or meaningless conversations was described.

“I told my parents that I can't let go of the stress. What I want is... I don't know... I guess it would be nice if they'd console me, but all they said was ‘take it easy'.” (Case 12)

“When dad was here, he'd ask how I was doing, and that's it. That was all we would say to each other.” (Case 13)

#### Enmeshment and over-involvement (n = 2)

Some participants described emotional enmeshment and over-involvement between their parents/caregivers and themselves. These close but insecure relationship patterns were unable to provide effective communication between parents/caregivers and children.

“In fact, all my mom has is me, and all I have is her. She's concerned about everything.”

(When the interviewer asked for the reason why she did not communicate her difficulties with her mother, even though she had close relationship with her mother) “I didn't want my mom to worry about me. I was afraid that she wouldn't understand.” (Case 13)

### Factors related to the cessation of intentional self-harm

#### Internal factors

##### Negative perception of self-harm and desire to stop (*n* = 11)

After self-harming for a period of time, several participants subsequently developed negative perceptions and attitudes toward self-harm. For example, they believed self-harm was useless or caused more trouble for themselves and their parents. Some participants described negative emotional reactions of their parents when the self-harm was disclosed. These insights motivated some participants to stop self-harm behaviors.

“I feel like hurting myself doesn't help much in the end.” (Case 6)

“I used to think the more I hurt myself, the better I would feel. But in fact, it only made me worse.” (Case 11)

“There are scars, permanently with me. When I started do it, it seemed to be addicted. Kept doing. Then, others knew and my mom saw the scene. She was sad.” (Case 13)

##### Increase of adaptive coping (*n* = 8)

A variety of coping strategies were used among participants to cope with self-harm. For instance, using music and social media as emotional coping, intentional distraction themselves from self-harm to other activities, and having more conversation or verbal expression with other people.

“When I'm busy with something else, I don't think about these things (self-harm).” (Case 9)

“It gets easier for me to talk about this (feeling) with other people.” (Case 11)

##### Finding life purposes (*n* = 6)

Some participants referred to the moments when they gained new insights regarding their purposes to live or the meaning of their lives. The doubt about the reasons to live and the meaning and values of their lives which was followed by the feeling of emptiness and other negative feelings often precipitated acts of self-harm. As a result, it became a relief after they found the purpose of their lives.

“I tried to stop as soon as I realized that it didn't matter if no one loves me. I can have a good life. I can build my own future, the kind that I want, have a family that I'd like to have.” (Case 9)

“I have a sister I need to care for. If I'm gone, then who's going to take care of her?” (Case 20)

##### Improvement of psychiatric symptoms (*n* = 1)

One participant described the direct association between the improvement of psychiatric symptoms and the decreasing self-harming behaviors.

“The feeling (urges to self-harm) just faded by itself after a while. It didn't reach the peak like it did before.” (Case 17)

### External factors

#### Supportive relationships and verbalization (*n* = 13)

Most of the participants mentioned that they stopped self-harm because of some supportive relationships, including relatives, friends, teachers, mentors, and lovers. These relationships provided not only psychological support but also the person whom the participants could ventilate with and verbalize their feelings and difficulties. Moreover, some participants clearly described that verbalization was used instead of acting out.

“I have many good people around. People who care. I have lots of friends and they are all nice. So, it's like I do matter to someone.” (Case 7)

“Some of my male friends reached out and supported me. They were good to me and so I got better.” (Case 9)

***If you have a chance to give suggestions to people who has self-harming, what are your suggestions? (Interviewer)***

“Find the one we most trust. Better to talk. Let words express, instead of action.” (Case 8)

“Find people you can ventilate. Because sometimes feeling pain in your heart and when we can ventilate, we don't want to self-harm.” (Case 10)

#### Treatments/interventions (n = 10)

The psychiatric treatments, including medications, doctor–patient relationships, psychoeducation for parents/family members, and other psychosocial interventions, showed therapeutic effects in some participants as they described.

“This doctor prescribed me antidepressants which helped a lot. He is also very nice and actually cares. Other doctors I'd seen before only asked things and that was it. It was barely a conversation; unlike with this doctor.” (Case 14)

“Ever since my parents got to talk to the doctor, they tried to understand more.” (Case 16)

#### Unwanted consequences of self–harm (n = 6)

After self-harming, some participants faced unwanted consequences such as misunderstandings with friends and negative reactions from significant others. These consequences negatively affected the participants more than self-harm *per se*.

“I decreased because I didn't want my friends to be afraid of me.” (Case 9)

“I guess it was because it made my mom sad, so I quitted.” (Case 13)

“I don't want my friends to think I'm calling for attention.” (Case 19)

#### Situations related to positive feelings (n = 6)

Occasionally, some situations linked with positive feelings helped the participants to experience their lives differently from the ways they used to. These experiences also facilitated new perceptions of themselves, their lives, and others. Moreover, most experiences obviously promoted social interactions such as chorus singing and outdoor activities with friends.

“There are small kids (relatives) around. Playing with them makes me really happy.” (Case 4)

“One of the happy times I can think of is when I go to church. I like going to church. I enjoy singing there.” (Case 18)

#### Behavioral control (n = 3)

Three participants reported that behavioral control by parents and teachers, such as limiting access to means of self-harm, caused reduced self-harming behaviors.

“They got rid of every single tool (for self-harm). I thought I hid it, but somehow, they found it and took it anyway.” (Case 20)

## Discussion

The recruitment of 20 adolescents and young adults with a history of intentional self-harm in this present study revealed six themes regarding predisposing child-rearing environments and nine themes regarding factors related to the cessation of intentional self-harm. In addition, most participants reported multiple functions of self-harm; however, intrapersonal functions were predominantly demonstrated as all participants reported that they used self-harm as an intrapersonal strategy for emotional regulation. Focusing on the development of self-harming behaviors, child-rearing environments interplayed with various types of psychosocial adversity, such as maltreatment and bullying, tended to predispose the acts of self-harm. Moreover, various internal and external factors related to the cessation of intentional self-harm were demonstrated in this present study.

Emotional availability (EA) refers to the capacity of the dyad (caregiver–child) to share an emotionally healthy relationship which includes six components: four caregiver and two child components (Biringen et al., 2014). The caregiver components are sensitivity, structuring, non-intrusiveness, and non-hostility. The adult sensitivity component refers to the ability to have a clear perception of the child's emotional expressions and provide appropriate parental responsiveness. According to this study population, most participants (*n* = 13, 65%) described their experience regarding the lack of emotional responsiveness or emotional neglect. In addition, some participants (*n* = 4, 20%) mentioned the superficial responsiveness of their caregivers or family members. Therefore, young people with a history of intentional self-harm tended to have predisposing experiences of poor emotional sensitivity and responsiveness in their upbringing environment. Moreover, the non-hostility component of EA tended to be low in participants who reported negativity, criticism, and harsh punishment in their interactions with caregivers. In addition, a non-intrusiveness component of EA, which refers to qualities such as lack of interference and over-protection, tended to be low in participants who reported enmeshment and over-involvement between their parents/caregivers and themselves. In general, caregiver components of EA in many participants tended to be poor; however, the interplay between caregiver components and child components, which are the child's responsiveness to the caregiver and the child's involvement in the caregiver, should be considered. The finding of this present study supports the link between intentional self-harm and poor emotional development. Congruently, Tao et al. (2020) conducted the study, which included 662 junior high school students in China. They found that father–child and mother–child attachment of the students could both directly and negatively influence self-injury behaviors. Moreover, this study demonstrated negative emotion and emotional coping styles as mediators in mother–child and father–child attachment models.

However, understanding the importance of emotional development and interpersonal and intrapersonal factors is not enough to explain the complexity of the phenomenon of intentional self-harm. Several sociocultural factors might involve with this phenomenon. In this present study, we found some participants reported high academic expectations originating from their families associated with their self-harming behaviors. The evidence showed that the parents' own backgrounds, hopes, and expectations could influence their children's future occupations (Irwin and Elley, 2013). In addition, stress on academic performance has been common in the educational system in Thailand and other Asian countries (Tan and Yates, 2011). For example, admission to high-quality schools/universities is a highly competitive situation in Thailand. As a result, high academic expectations as sources of stress tended to stimulate the emotional system and precipitate self-harming behaviors.

In recent years, a few studies focused on how people with intentional self-harm stop their behaviors. In 2017, Mummé et al. (2017) conducted a systematic review that included eight studies regarding the cessation of non-suicidal self-injury. They reported both intrapersonal and interpersonal factors influenced the cessation of self-harm, including family support, self-esteem, emotional regulation, and professional help. In this study, we similarly found supportive relationships, both from families and non-families, and professional treatments were parts of the cessation processes. Moreover, we demonstrated that verbalization in a safe and supportive atmosphere was an important process that helped several participants feel relieved and promoted emotional regulation. Some participants clearly described that verbalization was used instead of acting out self-harm. In addition, we similarly demonstrated the increase in adaptive coping, especially for emotional regulation or emotional coping, related to the cessation of self-harm. On the other hand, we did not demonstrate that self-esteem was directly associated with the cessation of self-harm. However, we found the situations related to positive feelings, which were probably related to self-esteem associated with the cessation of self-harm. We hypothesized that the increase in self-esteem, self-worth, and self-acceptance might embed in these positive situations.

In addition, Brennan et al. (2022) conducted a systematic review and a meta-synthesis of 56 studies regarding what helps people to reduce or stop self-harm. They reported two meta-themes that were breaking the chain of self-harm and building a new foundation for change. First, breaking the chain emphasizes the immediate strategy to break the link between a person's current psychological or social state and the act of self-harm. At the same time, they were building a new foundation for change, referred to a longer-term strategy and actions that strengthened the separation between self-harm and a person's way of life, including arriving at a positive view of self, reassessing place in the social world, and re-orienting to a more positive future. Congruently, this study described finding life purposes as an internal factor related to the cessation of self-harm. It tended to be one way to re-orient to a more positive future, as reported by Brennan et al. (2022). Moreover, Rissanen et al. (2013) studied the factors contributing to the cessation of self-cutting among 13–18-year-old Finnish adolescents by using self-rating questionnaires and asking the participants to write their own descriptions of how they had been able to stop self-cutting. Realizing uselessness, irrationality, stupidity, unhelpfulness, and unattractiveness of self-cutting was reported as the factors associated with self-cutting which contributed to the cessation of self-cutting. Consistently, this study reported negative perceptions of self-harm, such as uselessness and unhelpfulness, and unwanted consequences of self–harm as factors related to the cessation of self-harm. It was possible that young people who lacked experience and lacked skills for emotional regulation tried to use self-harm as a solution for their emotional disturbance; however, they consequently found the opposite side of this strategy.

The strengths of this study included qualitative research methods which emphasized on human experiences and provided the understanding and rationale behind participants' behaviors. As a result, this study supported the link between intentional self-harm and poor emotional development embedded in predisposing parent–child interactions and child-rearing environments. In addition, the in-depth interviews in this present study performed by experienced clinicians included both general psychiatrists and child psychiatrists. Therefore, the sensitive study population was professionally handled by experienced clinicians, and sensitive issues such as abusive history, violence, and emotional disturbance could be appropriately explored. Moreover, developmental framework and perspectives were applied in this study; therefore, the study population included both adolescents and young adults as a continuum. However, this study has several limitations. First, the study population was a clinical population recruited from tertiary care; moreover, it was predominately female. This specific study population might affect the generalizability of this study. Second, sociocultural factors demonstrated in this study might be unable to apply to other societies; however, they might be similar and might be applied in some societies, such as societies with high academic competition. Finally, the duration of the self-harming cessation defined in this study was quite short (1 month) because we intended to focus on the starting points of cessation or decrease of self-harming behaviors. Therefore, the results from this study might not demonstrate the permanent cessation of these self-harming behaviors.

## Conclusion

This qualitative study revealed six themes regarding predisposing child-rearing environments and nine themes regarding factors related to the cessation of intentional self-harm. This study underscored the importance of viewing self-harm as a complex phenomenon, and it is essential to understand the developmental pathways and the pathways to the cessation of these complex behaviors. Moreover, various internal and external factors related to the cessation of intentional self-harm were demonstrated, and verbalization in a safe and supportive atmosphere tended to be an important process to promote the cessation or decrease of intentional self-harm.

## Data availability statement

The original contributions presented in the study are included in the article/supplementary material, further inquiries can be directed to the corresponding author.

## Ethics statement

The studies involving human participants were reviewed and approved by the Ethics Committee of Ramathibodi Hospital, Mahidol University. Written informed consent to participate in this study was provided by the participants' legal guardian/next of kin.

## Author contributions

NL: planned and conducted the study, interviewing, coding, thematic analysis, drafted, and revised the final manuscript. AL: conducted and managed the study, interviewing, coding, thematic analysis, and read the manuscript. TP: planned the study, interviewing, coding, thematic analysis, and revised the final manuscript. TT: interviewing, thematic analysis consultation, and revised the final manuscript. PL, MT, and PW: interviewing and coding. All authors contributed to the article and approved the submitted version.
